# Role of meningeal immunity in brain function and protection against pathogens

**DOI:** 10.1186/s12950-023-00374-7

**Published:** 2024-01-30

**Authors:** Julie Rebejac, Elisa Eme-Scolan, Rejane Rua

**Affiliations:** Centre d’Immunologie de Marseille-Luminy, Aix Marseille Université, Inserm, CNRS, Marseille, France

**Keywords:** Meninges, Central nervous system, Infections, Meningitis, Cognition

## Abstract

The brain and spinal cord collectively referred to as the Central Nervous System (CNS) are protected by the blood-brain barrier that limits molecular, microbial and immunological trafficking. However, in the last decade, many studies have emphasized the protective role of ‘border regions’ at the surface of the CNS which are highly immunologically active, in contrast with the CNS parenchyma. In the steady-state, lymphoid and myeloid cells residing in the cranial meninges can affect brain function and behavior. Upon infection, they provide a first layer of protection against microbial neuroinvasion. The maturation of border sites over time enables more effective brain protection in adults as compared to neonates. Here, we provide a comprehensive update on the meningeal immune system and its role in physiological brain function and protection against infectious agents.

## Background

Microglia are the primary phagocytes of the CNS parenchyma and are involved in both homeostatic function and disease (reviewed in [1]). However, it is now proven that other immune cells exist at the brain borders and play a role in homeostasis and inflammation. The sites located at the interface between the parenchyma and the periphery include the perivascular spaces and the choroid plexus which have been reviewed elsewhere [2] as well as the meninges, the focus of this review. The meninges enveloping the CNS consist of three layers. The most superficial layer, the dura mater, is attached to the skull and contains a dense network of fenestrated blood vessels, lymphatic vessels, fibroblasts, immune cells, and nerves. The arachnoid mater is found under the dura, is avascular, and its epithelial cells are connected by tight junctions, forming a restrictive barrier. The subarachnoid space is filled with the cerebrospinal fluid (CSF) produced by the choroid plexus [3], which brings nutrients, trophic factors and collects waste of the brain [4]. Finally, the pia mater, the most inner layer, is composed of fibroblasts and endothelium also connected by tight junctions and sits on the glia limitans of the brain parenchyma [5]. Interestingly, fibroblasts from the three layers are transcriptionally distinct, suggesting specialized roles of those layers [6].

Unlike other tissues of the body, the brain parenchyma itself is devoid of lymphatic vessels. Draining is thus thought to be performed according to the “glymphatics” theory: the CSF collected in the periarterial space is pushed into the brain tissue by the pulsation of the arterioles and it then flows through water channels (aquaporin 4) towards the perivenous space. This enables the removal of waste from the brain [7]. The drainage then continues via the dural sinuses to the bloodstream, as well as the dural lymphatics vessels towards the deep cervical lymph nodes [8–10]. Another suggested route of exit of the CSF is through the nasal lymphatics draining the olfactory bulb [11]. The extent to which both pathways contribute to the drainage of the CSF remains a subject of ongoing debates.

The exploration of meningeal immunity is a nascent field which benefits from studies using cutting-edge technologies, such as CYTOF [12, 13] and single-cell RNA sequencing [14–20]. The dura mater is seeded by resident macrophages (the most abundant immune population), monocytes, dendritic cells, mast cells, innate lymphoid cells, B and T cells [13, 21]. Dural sinuses are immune hubs where T cells and antigen-presenting cells interact [17]. T cells traffic from the blood to the dural meninges, and drain to the deep cervical lymph nodes [22]. B cells mainly seed the meninges from the bone marrow but can also come from the blood [16, 23]. Both lymphoid populations accumulate in the meninges upon aging [13, 16] and their role in neurodegenerative diseases is reviewed elsewhere [24]. The leptomeninges, less permissive, primarily harbors resident macrophages and dendritic cells as well as few lymphoid cells [14, 21].

Interestingly, a two-way exchange between the skull bone marrow and the CSF has been described. The CSF can reach skull bone marrow upwards via dura-skull channels and on the opposite, immune cells can seed the meninges directly from skull [25–28].

In this review, we will delve into the functions of meningeal immune cells in both homeostasis and infections.

## Role of meningeal immunity on homeostatic brain functions

### Lymphoid cells (innate, innate-like and adaptive)

Evidence suggests that, under normal conditions, immune cells are present in the meninges and exert an influence on cognition without penetrating the brain parenchyma. Dural meninges contain ILC1/NK cells that produce both IFNγ and acetylcholine (Ach). Systemic depletion of ILC1/NK cells (using anti-NK1.1 depleting antibody) or of IFN-γ (using anti-IFNγ neutralizing antibody) decreases miniature inhibitory postsynaptic currents (mIPSCs) in the prefrontal cortex (PFC) [29]. This correlates with decreased memory in mice [29]. Furthermore, anti-NK1.1 treated mice have less Ach in the hypothalamus and less dopamine in the hippocampus, which correlates with decreased vigilance behavior. Direct Ach administration in the hypothalamus of anti-NK1.1 treated mice restores normal behavior. This suggests that NK/ILC1 promote both memory and vigilance behavior.

In addition, in Mr1^−/−^ mice lacking mucosal-associated invariant T (MAIT) cells, reactive oxygen species (ROS) accumulate in the dura and leptomeninges, which correlates with barrier dysfunction and memory impairment, observable in Y-maze and water maze tests [30]. It is worth mentioning that younger mice did not exhibit any cognitive impairment, raising the question of whether compensatory mechanisms present in early life are lost with aging. Other innate-like T cells are also involved in cognition. Indeed, loss of γδ-T cells or IL17, using TCRδ^−/−^ and IL17^−/−^ mice respectively, or intrathecal administration of anti-IL17 antibody, impair memory [31] or vigilance [32], although those studies could not reproduce each other’s findings. Of note, the mice models used are not specific to the dura and should not exclude the role of γδ-T cells or IL17 of peripheral organs on behavior.

Regarding adaptive lymphocytes, systemic blockade of CD4 + T cells using CD4 depleting antibodies reduces brain-derived neurotrophic factor (BDNF) production and hippocampal neurogenesis, which is associated with memory deficits [33, 34]. In addition, dural meningeal CD4 + T cells produce IL4 at steady-state and both IL4^−/−^ mice and IL4 receptor silencing on GABAergic neurons of the hippocampus and the retrosplenial cortex recapitulate memory impairment [35, 36]. Dural meningeal CD4 + T cells also produce IFNγ [37]. Interestingly, sociability in mice is decreased upon inhibition of T cells extravasation (using anti-VLA4 antibody), upon intrathecal administration of anti-IFNγ antibody and upon genetic ablation of Ifngr1 on PFC neurons, which also reduce inhibitory currents in the PFC [37]. This suggests that T-cell derived IL4 and IFNγ are involved in memory and sociability respectively.

Intriguingly, B cells and their progenitors are also detectable in the dura during steady-state conditions, where they undergo negative selection due to CNS-specific antigens [15, 16, 18]. Upon aging, peripheral B cells accumulate in the meninges [16]. B cells can affect behavior probably by regulating meningeal myeloid cell activation as Cd19^−/−^ mice have more meningeal neutrophils, activated monocytes and less exploratory behavior in the novel arena test [38]. Interestingly, chronically stressed mice had less dural and leptomeningeal B cells although the mechanism by which they decrease is still to be determined. This unveils an interesting correlation between stress and anxiety for which the link could be meningeal immunity.

**Fig. 1:**
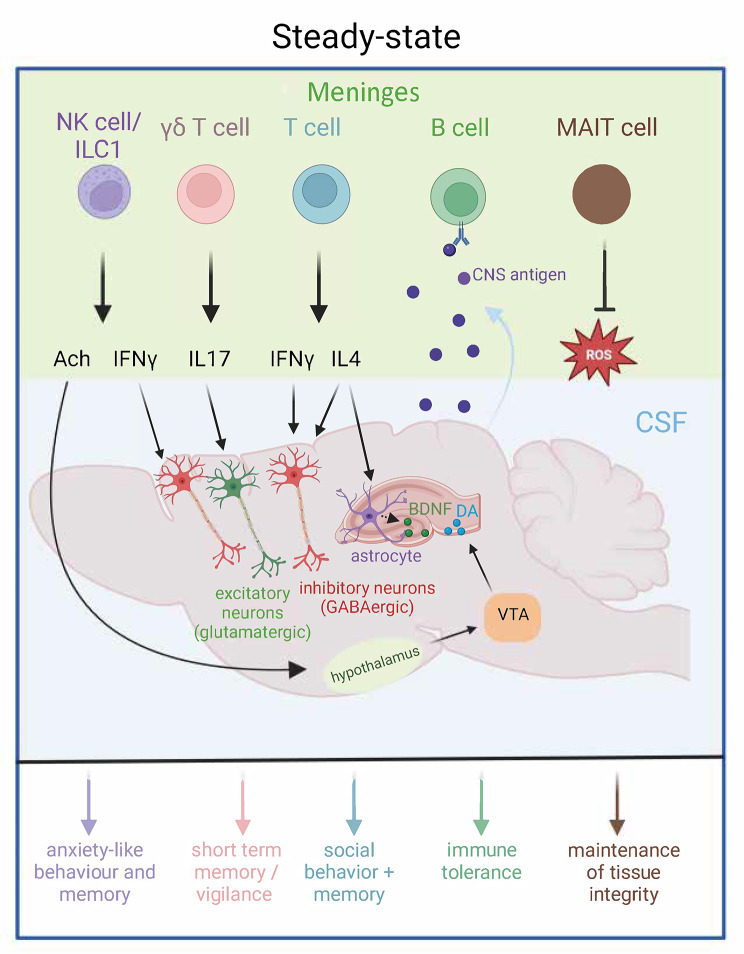
Simplified schematic view of meningeal lymphoid immune cells with established roles in cognition, behavior and CNS tolerance. In steady-state, immune cytokines secreted by meningeal immune cells can act upon brain cells and influence behavior and cognition. Some cytokines can directly exert their effect by binding on the receptors expressed by neurons (IL17, IFNγ and IL4). The precise mechanisms by which they cross the reach the parenchyma is still unknown. CNS antigens are drained from the subarachnoid space to the dura where they potentially interact with dural B cells. Black arrows indicate the origin and target of each cytokine and colored arrows resume the resulting functions. NK: natural killer; ILC1: innate lymphoid cell type 1; MAIT: mucosal-associated invariant T; Ach: acetylcholine; IFN: interferon; IL17: interleukin 17; IL4: interleukin 4; CNS: central nervous system; ROS: reactive oxygen species; CSF: cerebrospinal fluid; BDNF: brain-derived neurotrophic factor; DA: dopamine; VTA: ventral tegmental area

In sum, those studies underscore the presence of lymphocytes in the steady-state meninges and their potential to influence cognition and behavior, as illustrated in Fig. 1.

### Myeloid cells

Myeloid immune populations inhabit the meninges and border regions, particularly macrophages [12, 13, 18, 21, 39]. Recent advances in genetic and experimental approaches now allow for the separate examination of border cells versus parenchymal cells. The latter population can be studied using Tmem119-creERT2 [40], P2ry12-creER [41], Cx3cr1-ccre: Sall1-ncre [42] and Hexb-creERT2 [43] among other models. On the other hand, the models Cx3cr1-ccre: Lyve1-ncre [42], Cd163-cre [19], Pf4-Cre [44] and Mrc1-creERT2 [43] can target border macrophages (Table 1). In particular, meningeal macrophages (MM) can be categorized into two subpopulations based on their expression of major histocompatibility complex class-II (MHC-II). Interestingly, MHC-II expression increases with age [13, 19, 21, 45]. This elevation can be attributed, in part, to the influx of monocytes, maturation of MHC-II macrophages, presence of T cells and microbiota although additional factors such as infections can also exert an influence [21, 46]. Functionally, in vitro IL4-activated macrophages injected systemically but not intrathecally are shown to boost memory of severe immunodeficient mice (SCID). These mice show more IL10 expression in meningeal myeloid cells, suggesting a communication between peripheral cells and endogenous meningeal macrophages [47]. Of note, timing and location matters when considering macrophages’ cognitive functions, as in the absence of Smad4 during development, microglia adopt a transcriptomic profile similar to BAMs (subdural and perivascular macrophages), which results in impaired memory and more exploratory behavior [48]. Lastly, depletion of parenchymal border macrophages reduces extracellular matrix proteins degradation and CSF flow [49]. Overall, myeloid cells in the brain borders have specific signatures and their activation state influences cognitive performance.

**Table 1:** Summary of the strengths and weaknesses of different mice models used to target microglia or border macrophages relative to their recombination efficiency using reporter mice. The data provided from the studies are either based on flow cytometry (FC) or immunofluorescence (IF) assays and this is precised for each point

Model (line x reporter mouse used)	Strengths	Weaknesses
Tmem119-creERT2 x R26-TdT	• ~ 98% microglial recombination in adult and neonatal day 2 mice (IF/FC) • Small to no recombination observed in PVM, pial and CP macrophages (FC/IF) • No recombination in other parenchymal cell types (oligodendrocytes, astrocytes or neurons; IF).	• Small off-target effect on endothelium and pial cells • 3% blood leukocytes still tagged 7 days after treatment.
P2ry12-creER x R26-TdT	• ~ 94% microglial recombination in adult (IF/FC) • No effect on pial macrophages and PVM (IF). • No recombination in other brain cell types (oligodendrocytes, astrocytes or neurons; IF). • Limited off-target in other tissue macrophages and blood (IF/FC).	• 20% off-target effect on dural and CP macrophages (IF). • 40% CP macrophages targeted when induced in embryonic day 13.5 (IF).
Cx3cr1-ccre: Sall1-ncre x R26-TdT	• ~ 88% recombination in microglia when the split-cre is homozygous (FC). No target of dural, pial, CP macrophages (IF) and other organs (IF/FC).	• 23% recombination in microglia in Cx3cr1-ccre: Sall1-ncre heterozygotes (FC).
Hexb-creERT2 x R26-YFP	• ~ 90% of microglial recombination observed in adults (FC/IF). • Low recombination in PVM and subdural macrophages (0%), other brain cells, blood and other tissues (FC/IF).	• 60% off-target in the kidney if induced in adult (FC). • No observation of the dura.
Cx3cr1-ccre: Lyve1-ncre x R26-TdT	• 60% recombination pial macrophage in Lyve1 homozygotes (IF). • No recombination in choroid plexus macrophages (Lyve1-) (IF)	• 20% recombination in Lyve1 heterozygotes (IF). • Recombination in some organs (heart, lung, adipose tissue) (FC/IF) • Unknown effectiveness in the dura mater (some recombination observable but not quantified).
Cd163-cre x R26-YFP	• Recombination of 70% of dural macrophages and around 50–60% of brain macrophages (pial/CP/PVM) (FC) • No off-target effect on the microglia (FC)	• Not specific to the CNS compartment (other peripheral organs express CD163)
Mrc1-creERT2 x R26-TdT	• ~ 95% PVM and pial macrophages targeted • No recombination in other brain cell types (IF; oligodendrocytes, astrocytes or neurons), blood immune cells (FC). • Heterozygotes and homozygotes have similar efficiency of recombination	• No observation of the dura
Pf4-Cre x R26-TdT	• 100% of perivascular, pial and dural macrophages (IF) and 80% of CP macrophages. • ~ 5% microglia targeted (FC).	• Recombination in a neuronal population in the anterior amygdala and in other organs and platelets.

## Role of meningeal immunity in adult CNS infections

### Lymphoid cells

Meningeal lymphocytes also play a role in CNS infections. First, CD8 + T cells are recruited in the dura and pia mater and are important to reduce viral load upon intracranial lymphocytic choriomeningitis virus (LCMV) infection [50]. However, they also recruit monocytes and neutrophils which promote brain edema and herniation [51].

In parallel, during a parasitic infection by *Toxoplasma gondii*, a recruitment of regulatory T cells (Tregs) is observed in the meninges and perivascular spaces, where they interact with CD11c + cells. The authors hypothesize that these interactions allow Tregs to limit the activity of dendritic cells, therefore the effector T cell responses [52]. As suggested by the authors this could limit the entry of Tregs in the brain parenchyma, strengthen the action of effector T cells against the pathogen.

Interestingly, meninges also contain resident B cells (IgA + plasma cells) concentrated around the dural venous sinuses. Local depletion of those cells during peripheral fungal infection by *Candida albicans* leads to the invasion of the brain parenchyma by the pathogen [23]. Curiously, those IgA + plasma cells shared B cell receptor sequences with the intestine resident population, and were depending on microbiota. We can therefore wonder about the evolutive interest of having gut-derived plasma cells in the meninges.

### Myeloid cells

Myeloid cells are key players in the regulation of infection and inflammation. In case of viral infection, for instance following intracranial LCMV injection, meningeal macrophages derived from blood monocytes engraft the tissue and partially replace resident ones, which affects the response to a secondary challenge due to their low reactivity to acetylcholine and LPS [46]. In parallel, our team showed that MM prevent neuroinvasion and fatal meningitis upon LCMV peripheral infection [19]. When MM are chemically or genetically depleted, or when these cells are unable to respond to IFN-I signaling, mice experience fatal meningitis, whereas the control group of mice survive. In addition, by measuring viral RNA in dural MM populations over time, we found that MHC-II + MM are more efficient at clearing the virus than MHC-II- MM [19].

In addition, peripheral parasitic infection by *Trypanosoma brucei* (the agent of sleeping sickness), leads to a massive infiltration of peripheral monocytes in the meninges (dura and sub-dural) and choroid plexus, that afterward differentiate towards macrophages. Those monocytes-derived macrophages show severe transcriptional changes, which divert from the ones of resident-macrophages. Interestingly, transcriptomic changes are more sustained in border macrophages, even though pathogens are also invading the brain parenchyma, suggesting that neuroinfection can leave a stronger mark on border cells [53]. Upon infection of Cx3cr1-creER:R26-DTR mice by *Trypanosoma brucei*, the depletion of resident macrophages within two weeks is associated with an increased parasite load and immune infiltrate in the dura. However, depletion at a later stage, once the parasites have invaded the choroid plexus and CSF, does not impact the parasite numbers. This could be explained by a functional redundancy of the resident and infiltrated immune cells [53].

Moreover, in a model of intravenous bacterial infection with *Streptococcus agalactiae* or *Streptococcus pneumoniae*, a communication between meningeal nociceptors and dural MM is observed. Indeed, dural nociceptors activated by the bacteria are secreting the neuropeptide Calcitonin Gene-Related Peptide (CGRP) which is detected by MM and blocks their chemokine secretion and antibacterial action [44]. Furthermore, depletion of macrophages in the dura (and probably the pia) using an intracisternal injection of clodronate-laden liposomes leads to reduced immune recruitment to the dura while bacterial load is increased in the dura and consequently in the brain parenchyma [44]. It is suggested that depletion of the dural (and pial) macrophages impairs the overall cytokine production from the meninges, and therefore impairs recruitment of immune cells, and higher leakage of bacteria in the parenchyma.

It is important to note that in all models studied (parasitic, viral or bacterial), depletion of meningeal macrophages leads to increased pathogen load in the brain.

### Non-immune cells

Meningeal non-immune cell populations also have a role in the protection of the CNS. Upon infection by the neurotropic mouse hepatitis virus (MHV), endothelial cells and fibroblasts of the leptomeninges recruit antiviral IFNγ-producing CD8 + T cells in the CNS, through the secretion of CCR7 ligands [54]. Moreover, dural lymphatic vessels mediate the drainage of intravenously injected Japanese encephalitis virus (JEV) present in the CNS toward the draining lymph nodes where it is presented by antigen-presenting cells likely promoting antiviral functions. Consequently, a surgical ligation of the afferent dural lympathics or their ablation using a visudyne and photoconversion-based approach, worsens viral load, inflammation and survival of the infected mice [55]. Opposingly, VEGF-C treatment, which promotes lymphatics development, ameliorates the survival of the JEV-infected mice [55]. Moreover, during viral infection of the CNS by JEV, a lymphangiogenesis is observed in the dura, while the lymphatic vessels function in the dura is impaired, suggesting a compensation mechanism [55].

Finally, in the case of a peripheral infection by the parasite *Toxoplasma gondii*, ligation of meningeal lymphatics leads to an activation of dendritic cells and CD4 + and CD8 + T cells, although this had no discernable effect on neuroinvasion [56]. Therefore, those studies suggest that meningeal lymphatic vessels are involved in the resolution of CNS infection, but their involvement remains to be defined and could be pathogen-specific.

**Fig. 2:**
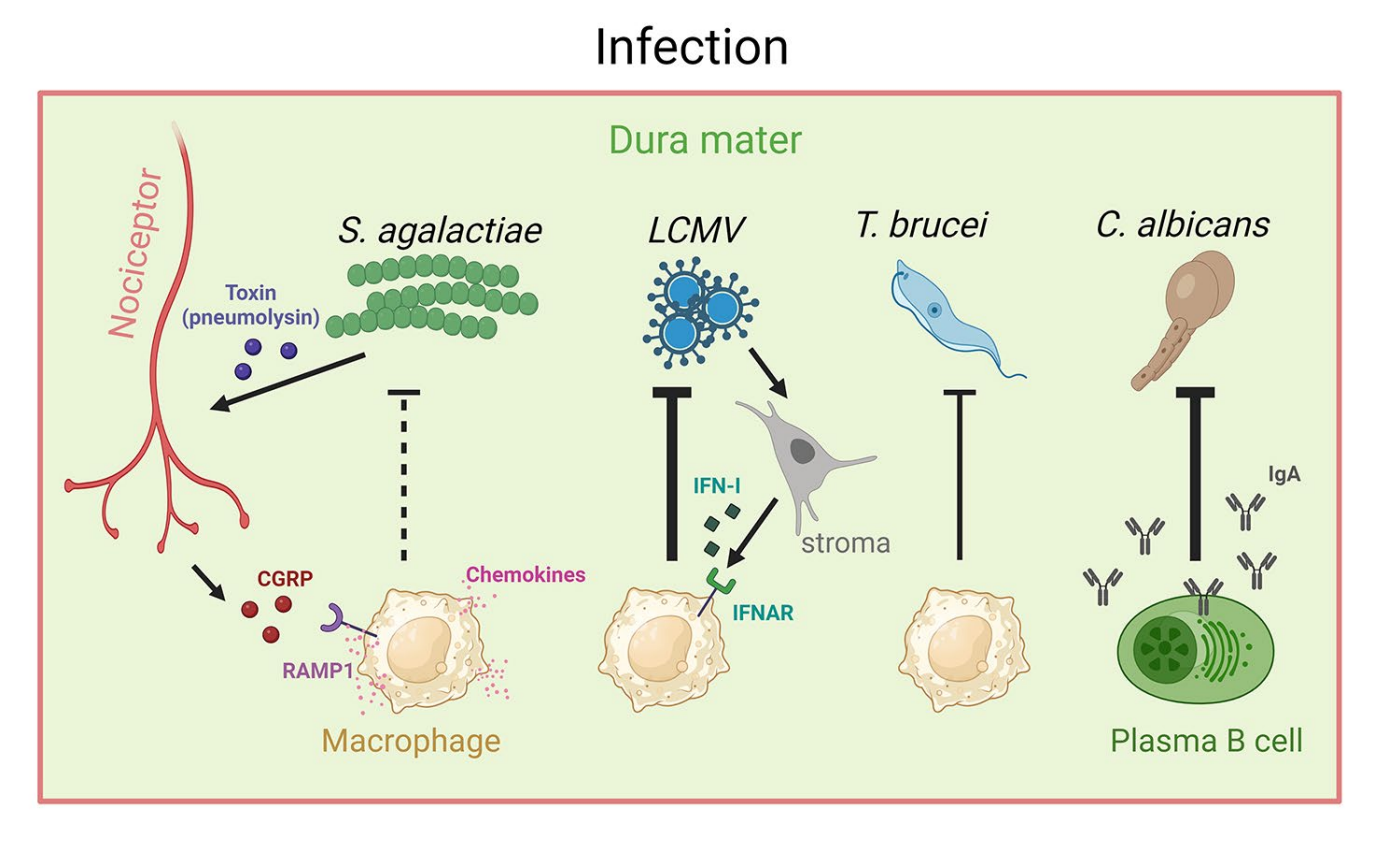
Schematic view of meningeal immune cells and their role in infection. In an infectious context, the meningeal immune response is important to fight pathogens and keep them from invading the brain. The arrow indicates the cellular target of the molecules/virus. Lines originating from immune cells and their thickness represent the strength of the defense provided by the immune cells. S. agalactiae: *Streptococcus agalactiae*; LCMV: lymphocytic choriomeningitis virus; T. brucei: *Trypanosoma brucei*; C. albicans: *Candida albicans*; CGRP: calcitonin gene-related peptide; RAMP1: Receptor Activity Modifying Protein 1, IFNAR: interferon type 1 receptor; IgA: immunoglobulin A

In sum, those studies underscore the presence of meningeal immune cells in infectious settings and their potential to influence inflammation, as illustrated in Fig. 2.

## Role of meningeal immunity in neonatal CNS infections

Neonates show a high susceptibility to meningitis caused by hematogenous pathogens [57]. In the steady-state, neonatal pups display higher homeostatic Wnt activity in the intestine and the choroid plexus epithelium which may correlate with weaker tight junctions and more permissive barriers than adult mice [58]. Upon oral infection with *Streptococcus agalactiae*, the absence of mature microbiota and the permeability of the intestine and choroid plexus epithelium increases *Streptococcus* colonization of the gut, favoring systemic dissemination and meningitis [58]. Similarly, postnatal day 7 mice intraperitoneally inoculated with LCMV are more susceptible to fatal infection compared to adult mice. As opposed to immuno-competent adults in which the virus transiently infects the dura before being cleared by meningeal macrophages and CD8 + T cells, in neonatal mice the virus first invades the dural sinuses then propagates to the leptomeninges, choroid plexus and brain parenchyma [20]. The authors suggest that dural MHC-II + macrophages are more potent to respond against LCMV infection as previously suggested [19]. However, models specifically targeting this macrophage population are still missing to validate this hypothesis. To sum up, the incomplete formation of protective barriers and the underdeveloped immune system surrounding the brain hinders its ability to effectively combat infections in neonates.

## What about the skull?

Immune cells can be recruited to the meninges from the blood vessels but also from the overlying skull bone marrow. Indeed, direct channels connecting the skull bone marrow to the meninges have recently been discovered, and they allow a CNS-skull communication [25, 27, 28]. The transcriptomic signatures of neutrophils in the skull and the meninges share similarities suggesting their common origin [59], in agreement with previous studies indicating that the skull bone marrow is reservoir of immune cells for the meninges and the CNS [60]. Indeed, infiltration of cells from the skull bone marrow to the meninges has been observed in stroke [27], experimental autoimmune encephalomyelitis [60] and cancer [61]. The skull-meningeal communication is bidirectional, and can also function upwards. For instance, following intracerebroventricular injection of *Streptococcus pneumoniae*, bacteria were found in the meninges and reached the calvaria bone marrow through those bone channels. There, it induced cranial hematopoiesis [26]. Therefore, even though the contribution of skull in the overall defense of the CNS is not fully described for now, new studies will hopefully bring relevant information and concepts.

## Concluding remarks

Since the rediscovery of lymphatic vessels in the CNS [8], extensive work has been done to characterize immune and non-immune cells of the brain borders in steady-state and infection. Neuromodulation via immune cytokines can be found across species, from worms to humans [62]. Like in mice, human dura mater also contains antigen-presenting cells and can accumulate brain-derived proteins [17] suggesting shared features in both species. Nevertheless, there are still unresolved questions. For instance: (i) At rest, how do the cytokines from the dura gain access the subarachnoid space and subsequently cross the BBB? (ii) What different roles do the three layers of the meninges (dura, arachnoid pia) play? For now, the majority of studies have primarily focused on the dura. Future studies using new genetic tools would aid in differentiating the roles of each immune compartment. Overall, these discoveries on meningeal immunity shine a new light on strategies to treat brain diseases.

## Data Availability

Not applicable.

## Abbreviations

Ach: acetylcholine
BAM: barrier-associated macrophages
BBB: blood-brain barrier
BDNF: brain-derived neurotrophic factor
CGRP: calcitonin gene-related peptide
CNS: central nervous system
CSF: cerebrospinal fluid
CYTOF: cytometry by time of flight (mass cytometry)
EAE: experimental autoimmune encephalomyelitis
IFN-γ: interferonγ
ILC1: innate lymphoid cell type 1
JEV: Japanese encephalitis virus
LCMV: lymphocytic choriomeningitis virus
MAIT: mucosal-associated invariant T cell
MHC-II: major histocompatibility complex class IIMHV:mouse hepatitis virus
mIPSCs: miniature inhibitory postsynaptic currents
MM: meningeal macrophages
NK: natural killer
PFC: prefrontal cortex
RAMP1: Receptor Activity Modifying Protein 1
ROS: reactive oxygen species
VEGF-C: vascular endothelial growth factor C
